# Secretion of Acetylxylan Esterase From *Chlamydomonas reinhardtii* Enables Utilization of Lignocellulosic Biomass as a Carbon Source

**DOI:** 10.3389/fbioe.2019.00035

**Published:** 2019-02-28

**Authors:** Erick Miguel Ramos-Martinez, Lorenzo Fimognari, Maria K. Rasmussen, Yumiko Sakuragi

**Affiliations:** Department of Plant and Environmental Sciences, Faculty of Science, University of Copenhagen, Frederiksberg, Denmark

**Keywords:** *chlamydomonas reinhardtii*, protein secretion, acetylxylan esterase, lignocellulosic biomass, direct application of engineered algae

## Abstract

Microalgae offer a promising biological platform for sustainable biomanufacturing of a wide range of chemicals, pharmaceuticals, and fuels. The model microalga *Chlamydomonas reinhardtii* is thus far the most versatile algal chassis for bioengineering and can grow using atmospheric CO_2_ and organic carbons (e.g., acetate and pure cellulose). Ability to utilize renewable feedstock like lignocellulosic biomass as a carbon source could significantly accelerate microalgae-based productions, but this is yet to be demonstrated. We observed that *C. reinhardtii* was not able to heterotrophically grow using wheat straw, a common type of lignocellulosic biomass, likely due to the recalcitrant nature of the biomass. When the biomass was pretreated with alkaline, *C. reinhardtii* was able to grow using acetate that was released from the biomass. To establish an eco-friendly and self-sustained growth system, we engineered *C. reinhardtii* to secrete a fungal acetylxylan esterase (AXE) for hydrolysis of acetylesters in the lignocellulosic biomass. Two transgenic strains (CrAXE03 and CrAXE23) secreting an active AXE into culture media were isolated. Incubation of CrAXE03 with wheat straw resulted in an eight-fold increase in the algal cell counts with a concomitant decrease of biomass acetylester contents by 96%. The transgenic lines showed minor growth defects compared to the parental strain, indicating that secretion of the AXE protein imposes limited metabolic burden. The results presented here would open new opportunities for applying low-cost renewable feedstock, available in large amounts as agricultural and manufacturing by-products, for microalgal cultivation. Furthermore, acetylesters and acetate released from them, are well-known inhibitors in lignocellulosic biofuel productions; thus, direct application of the bioengineered microalga could be exploited for improving renewable biofuel productions.

## Introduction

Microalgae offer a promising biological platform for sustainable biomanufacturing of a wide range of bioproducts from water, air, and light. *C. reinhardtii* is one of the best characterized algal species and has been developed as a robust expression platform for production of recombinant proteins, oils, and chemicals with broad industrial applications (Almaraz-Delgado et al., 2014; Rasala and Mayfield, 2015). Well-characterized molecular tools are also available and allow efficient and robust expressions of transgenes from the nuclear and chloroplast genomes, making this microalga by far the most powerful chassis for algal synthetic biology and bioengineering (Schroda et al., 2000; Eichler-Stahlberg et al., 2009; Jinkerson and Jonikas, 2015; Mussgnug, 2015; Díaz-Santos et al., 2016).

*Chlamydomonas reinhardtii* can grow photoautotrophically using CO_2_ as the sole carbon source and also heterotrophically or mixotrophically using organic carbons such as acetate (Harris, 2009). Furthermore, it was recently shown that *C. reinhardtii* can utilize pure cellulose through secretion of cellulolytic enzymes (Blifernez-Klassen et al., 2012), raising an exciting prospect of using plant biomass, such as agricultural residues and manufacturing by-products, as alternative carbon sources for improving the cost performance of microalgae-based productions. However, direct utilization of renewable biomass by *C. reinhardtii* is yet to be demonstrated. Thus far, evidence of direct utilization of lignocellulosic biomass is only reported for the oleaginous microalga *Auxenochlorella protothecoides* UTEX 25 (Vogler et al., 2018).

Over 30 recombinant proteins have been produced in *C. reinhardtii*, a small subset of which were secreted to culture media. Recombinant protein secretion into culture media has several advantages, including protein glycosylation, which can faciliate folding and increased protein stability (Lingg et al., 2012; Mathieu-Rivet et al., 2014; Ramos-Martinez et al., 2017), and simplification of downstream processing, which can circumvent costly cell harvesting steps (Hellwig et al., 2004; Nikolov and Woodard, 2004). Moreover, the algal biomass can be exploited as a co-product, adding more values to the process (Gangl et al., 2015). Among secreted recombinant proteins produced by *C. reinhardii* are luciferase (Laursen et al., 2013), fluorescent proteins (Lauersen et al., 2015; Ramos-Martinez et al., 2017), xylanase (Rasala et al., 2012), laccase (Chiaiese et al., 2011), human glycoprotein erythropoietin (Eichler-Stahlberg et al., 2009), an ice-binding protein (Lauersen et al., 2013), and human growth factors (Chávez et al., 2016; Baier et al., 2018). Glyconengineering was recently applied to enhance the product yield (Ramos-Martinez et al., 2017). However, current yields are still very low, making further enhancement of product yields and product recovery technologies critically important (Baier et al., 2018). Alternatively, exploration into new avenues of applications, where engineered microalgal cultures could be directly utilized without product purifications, could open new opportunities, as it has been explored in yeast (Sun et al., 2012; Kricka et al., 2014; Liang et al., 2014; Lee et al., 2017).

In this study, we investigated utilization of lignocellulosic biomass for cultivation of *C. reinhardtii*. Lignocellulosic biomass is widely used as renewable feedstock for productions of bioethanol and animal feeds are available in huge amounts as agricultural residues such as wheat straw. Our initial test, however, revealed that *C. reinhardtii* was not able to grow using wheat straw as the carbon source. We exploited the fact that hemicelluloses and pectin in plant biomass can be highly acetyl esterified and it was previously shown that acetate released from softwood biomass could be assimilated by *C. reinhardtii*, although this required prior removal of toxic substances (Liang et al., 2013; Hu et al., 2016). To establish an eco-friendly and self-sustained system of biomass acetylester utilization, *C. reinhardtii* was engineered to secrete an acetylxylan esterase (AXE), capable of hydrolyzing acetylesters in lignocellulosic biomass. Our results demonstrated that the AXE-secreting *C. reinhardtii* was able to directly utilize acetylesters in lignocellulosic biomass, leading to simultaneous reduction in biomass acetylester contents.

## Materials and Methods

### Strains, Media, and Culture Conditions

*Chlamydomonas reinhardtii* wild type, photosynthetic mutant FUD16 (Ketchner et al., 1995), the cell wall-deficient strain UVM4 (Neupert et al., 2009), and transgenic strains generated in this study were routinely cultivated mixotrophically in tris-acetate-phosphate (TAP) media supplemented with 1 g L^−1^ acetic acid (Gorman and Levine, 1965) or in minimal medium (MM) containing 0.1 g L^−1^ acetic acid, instead of 1 g L^−1^ (Blifernez-Klassen et al., 2012). The pH value of all the media were adjusted to 7.0. Liquid cultures of *C. reinhardtii* were cultivated under the standard conditions (at the photon flux of 120 μmol m^−2^ s^−1^, 25°C) at a constant shaking (120 rpm) in an orbital shaker. Cell numbers in cultures were determined by counting in a Neubauer hemocytometer under a bright-field microscope. MM medium containing wheat biomass (MM+Biomass) was prepared as follows. Straws from wheat (*Triticum aestivum*) were first ground to small pieces, washed in 96% (v/v) ethanol followed by wash in 100% (v/v) acetone, and dried in air. Ten milligrams of dried straw were resuspended in 1 mL of a 50-mM ammonium formate buffer, pH 6.5, into which thermostable α-amylase (Megazyme, https://www.megazyme.com) was added at the final enzyme concentration of 2 U mL^−1^ to remove starch. The mixture was incubated for 2 h at 85°C and was centrifuged at 3,500 × g for 5 min at room temperature (~23°C). The pellet was washed in water and then in 100% (v/v) acetone and dried in air. The de-starched wheat straw was then added to MM at the concentration of 15 g L^−1^ and the mixture was autoclaved. The acetylester content of the de-starch biomass was 2.5% (w/w). All cultures were supplemented with antibiotics (50 μg mL^−1^ ampicillin, 25 μg mL^−1^ kasugamycin) to reduce the risk of contamination.

### Generation of the *CrAXE* Construct

All standard chemicals and reagents were purchased from SigmaAldrich (http://www.sigmaaldrich.com). Restriction enzymes were purchased from New England Biolabs (https://www.neb.com). Oligonucleotide primers were purchased from Integrated DNA Technologies Inc. (https://www.idtdna.com). The signal sequence in AXE protein from *Aspergillus nidulans* (AnAXE) was analyzed *in silico* analysis using the publicly available tool SignalP version 4.1 (http://www.cbs.dtu.dk/services/SignalP/). Codon optimization of the AXE gene was performed using the webtool OPTIMIZER (Puigbò et al., 2007). A codon-optimized AXE gene was synthesized (Integrated DNA Technologies, Inc.) and overhangs were added by PCR using primer AnAXEΔSP-F (5′-gcacagctcctgcgtgggccGTGAAGCTCCAGTACCTG) and AnAXE-R (5′-tcgaactgcgggtggctccaGCTCGTAAAACCGAACCAC). The uppercase letters indicate target specific sequences and the lowercase letters indicate overlapping sequences with a transformation vector (pERC-SSVENUS) (Ramos-Martinez et al., 2017). The PCR product was fused with the vector by overlap extension PCR (Bryksin and Matsumura, 2010) such that the codon-optimized *AXE* gene is in translational fusion with the gametolysin signal peptide (MSLATRRFGAAAALLVAACVLCTAPAWA) and a strep-tag (WSHPQFEK). The final expression gene construct was named CrAXE (Supplementary Material). The vector backbone contained the *aphVIII* gene encoding an aminoglycoside 3′-phosphotransferase from *Streptomyces rimosus* to provide resistance to paromomycin, which was used to select transformed *C. reinhardtii* (Sizova et al., 1996). The expression of the transgene and the resistance gene was controlled by the chimeric promoter *Hsp70A-RbcS2* containing the first intron of *RbscS2*. The *RbcS2* 3′ UTR was placed downstream of the transgenes and the resistance cassette for correct termination. For selection in *E. coli* the ampicillin resistance gene, *bla* encoding β-lactamase is introduced into the plasmid under the control of the *AmpR* promoter.

### Transformation of the UVM4 Strain of *C. reinhardtii*

The UVM4 strain was transformed by the glass bead method (Kindle, 1998) using 2 μg of the plasmid construct that has been linearized through *Sca*I restriction digestion. The mixture of the UVM4 cells and the linearized plasmid was incubated overnight and then harvested at 1,100 ×*g* for 5 min at room temperature (~23°C) and spread over TAP media containing 15 g L^−1^ agar and 15 mg L^−1^ paromomycin. After 7 days of incubation under the standard growth conditions, paromomycin resistant colonies were transferred to 96-well plates containing 250 μL of the liquid TAP media and 15 mg L^−1^ paromomycin in each well and were grown for 7 days. Ten microliters of each culture were transferred onto TAP agar media containing 15 mg L^−1^ paromomycin and further incubated under the standard conditions for 4 days. Each colony was transferred into 50 μL Chelex-100 5% (w/v) (Bio-Rad, https://www.bio-rad.com) in a well in 96-well PCR plate (VWR, https://www.vwr.com) and DNA was extracted as previously described (Cao et al., 2009). PCR was performed using gene specific primers (GSP3 5′-CTAGAACTAGTGCTGAGGCTTG and GSP4 5′-CGAAGGATCCCGCTTCAAATAC) and the HotMaster Taq DNA polymerase (ThermoFisher Scientific, https://www.thermofisher.com). PCR products were analyzed by gel electrophoresis in a tris-acetate-ethylenediaminetetraacetic acid buffer containing 1% (w/v) agarose.

### Reverse Transcriptase-PCR

Total RNAs were isolated from transgenic lines grown for 5 days in liquid TAP media until the cell density reached approximately 1 × 10^7^ cells mL^−1^ using the TRIzol reagent (Invitrogen, https://www.thermofisher.com) according to the manufacturer's instructions. Concentrations of purified RNAs were determined spectrophotometrically at 260 nm. One microgram of each sample was reverse transcribed into cDNA using iScript cDNA Synthesis kit (Bio-Rad) following the manufacturer's instruction. The following gene-specific forward primers were used: MR11 (5′-CTCATGCTGGACCGCCGC) and MR12 (5′-TTCTCGAACTGCGGGTGGCT). PCR was carried out using HotMaster Taq DNA Polymerase. PCR products were analyzed by gel electrophoresis as described above.

### Immunoblotting

The UVM4 strain and transgenic lines were grown for 5 days in TAP media under the standard conditions and 2 mL of each culture were centrifuged at 3,500 ×g for 5 min at the room temperature. The cell pellet was resuspended in 200 μL of water. The supernatant was transferred to a fresh tube and proteins were precipitated by mixing with 4 mL of pre-cooled acetone, followed by centrifugation at 12,000 ×*g* for 10 min at 4°C. The precipitate was air dried and dissolved in 200 μL of water. The resulting sample was mixed with four-fold concentrated Laemmlii Sample Buffer (Bio-Rad) in a 3:1 ratio and incubated at 95°C for 5 min. Forty five microliters of the mixture were loaded in a lane of a 12% Criterion™ TGX Stain-Free™ Protein Gel (Bio-Rad), and, after electrophoresis, proteins were transferred to a poly(vinylidene fluoride) membrane. Immunodetection of CrAXE was carried out using a strep-tag antibody (https://www.qiagen.com) and polyclonal rabbit anti-mouse antibodies conjugated with a horse-radish peroxidase (Dako, https://www.agilent.com). Probed proteins were visualized using the chemiluminescence substrate SuperSignal™ (ThermoFisher Scientific, Inc.).

### Acetylesterase Activity Assay

Microalgal strains were grown in liquid TAP media under the standard conditions. Acetylesterase activity was monitored as previously reported (Chung et al., 2002). One milliliter of each culture was centrifuged at 3,500 ×g for 5 min at the room temperature and 15 μL of the supernatant were transferred to a well in a 96-well plate. Subsequently, 285 μL of 100 mM potassium phosphate buffer, pH 7.0, containing 35 μM 4-nitrophenyl acetate was added. The mixture was incubated at 37°C and the changes in the absorbance at 410 nm was recorded using a Spectra MAX 190 plate reader (Molecular Devices, https://www.moleculardevices.com). One unit of enzyme activity is defined as the amount of enzyme catalyzing the hydrolysis of 1 μmol substrate per min per milliliter of sample.

### Determination of Biomass-Bound Acetylester Contents

Ten milligrams of the biomass materials were suspended in 1 mL of 1 M NaOH and incubated for 1 h at 22°C. The mixture was centrifuged at 3,500 ×g for 5 min and the supernatant was filtered through a 0.22-μm pore-size filter hydrophobic membrane. Acetic acid was detected by HPLC featuring a Synergi Hydro-RP (Phenomenex, http://www.phenomenex.com) column equipped with SecurityGuard Guard Cartridge (Phenomenex) and the diode-array detector SPD-M20A (Shimadzu, http://www.shimadzu.com). To elute analytes, 20 mM K_3_PO_4_, pH 2.9, was used at the constant flow rate of 0.7 mL min^−1^ at 22°C. Quantification was performed by integrating 210-nm peak area and comparing it with a standard curve generated using varying concentrations of acetic acid.

## Results

### Non-engineered *C. reinhardtii* Cannot Directly Utilize Wheat Biomass as the Carbon Source

**Figure 1:**
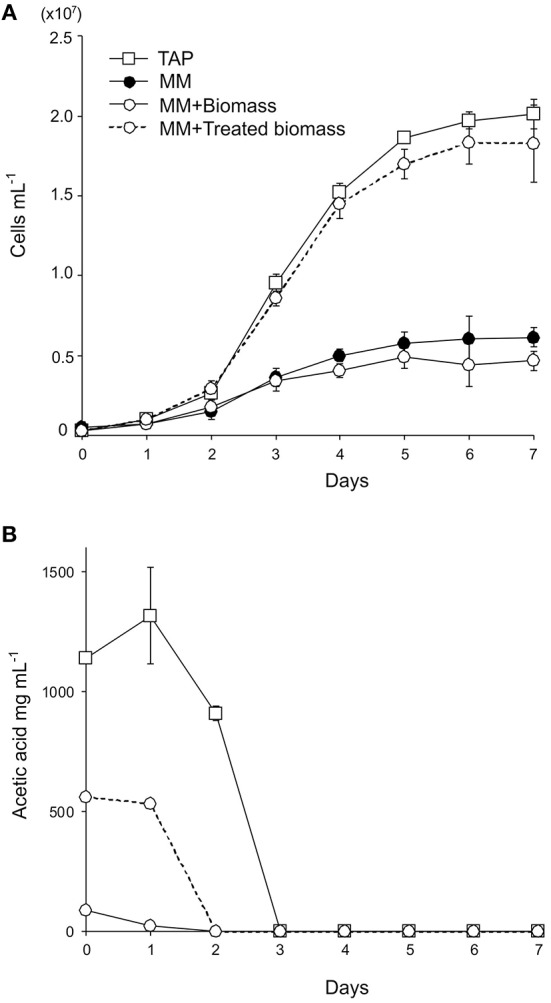
Heterotrophic cultivation of *C. reinhardtii* using wheat straw biomass. **(A)** Growth curves of the wild-type strain in enriched media (TAP media, square symbols with a solid line), minimal medium (MM, filled circles with a solid line), MM containing 15 g L^−1^ wheat straw biomass (MM+Biomass, open circles with a solid line), and MM containing 15 g L^−1^ alkaline-treated wheat straw biomass (MM+Treated biomass, open circles with a dotted line). **(B)** Acetate contents in the wild-type *C. reinhardtii* cultures in TAP, MM+Biomass, and MM+Treated biomass. Three independent cultures were analyzed for each conditions. Average values and standard deviations are shown.

**Figure 2:**
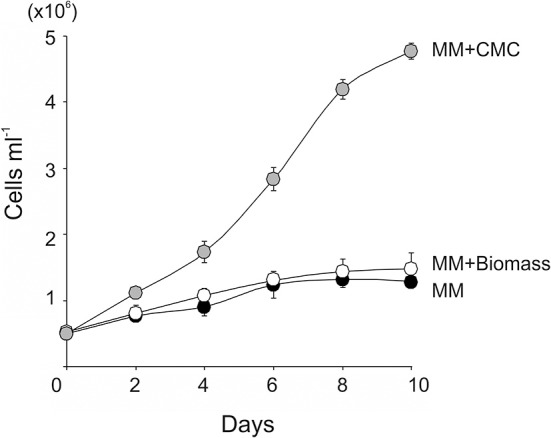
Growth of the photosynthesis deficient strain (FUD16) of *C. reinhardtii* using wheat biomass as a carbon source. The FUD16 strain was cultivated in the TAP, the MM containing 15 g L^−1^ of CMC (MM+CMC), and the MM containing 15 g L^−1^ of wheat biomass (MM+Biomass). Three independent cultures were analyzed for each condition. Average values and standard deviations are shown.

To test if lignocellulosic biomass can be used as a carbon source for *C. reinhardtii*, the wild-type *C. reinhardtii* was cultivated in minimal medium supplemented with 15 g l^−1^ of wheat straw biomass (MM+Biomass) in the dark. The wild type grew only slightly in MM+Biomass. No difference was observed between growth in MM+Biomass and that in MM (Figure 1A), indicating that wheat biomass did not support growth of the wild-type *C. reinhardtii* in the dark. Control experiments showed that the wild type grew robustly in TAP media in the dark. Next, the FUD16 mutant strain of *C. reinhardtii* was used to test heterotrophic growth using wheat biomass. This strain is defective in photoautotrophic growth and requires organic carbons for growth, thus allowing us to evaluate heterotrophic assimilation of wheat straw by the microalga (Blifernez-Klassen et al., 2012). Similar to the results described above, the FUD16 strain grew only slightly in MM and MM+Biomass. In contrast, when MM was supplemented with 15 g l^−1^ of carboxymethyl cellulose (MM+CMC), the FUD16 was able to grew as previously reported (Blifernez-Klassen et al., 2012) (Figure 2). These results indicate that *C. reinhardtii* is not capable of utilizing cellulose in lignocellulosic biomass as a carbon source, possibly because hemicelluloses and lignins occlude cellulolytic enzymes produced by the microalga.

Previously, wild-type *C. reinhardtii* was shown to assimilate acetate that was released from softwood following fast pyrolysis treatments (Liang et al., 2013; Hu et al., 2016). We tested if acetate released from wheat straw biomass can support growth of the microalga. Wheat biomass was treated with alkaline (1 M NaOH) and added to MM to generate “MM+Treated biomass” at the biomass content of 15 g l^−1^. The pH value of the media was adjusted to 7.0. The wild-type *C. reinhardtii* grew similarly well in MM+Treated biomass to that in TAP media (Figure 1B). Acetates in the culture media were completely removed by *C. reinhardtii* by day 3 of the cultivation, indicating that the wild-type *C. reinhardtii* can assimilate acetates chemically released from wheat biomass.

### Generation and Characterization of *C. reinhardtii* Secreting the CrAXE Enzyme

**Figure 3:**
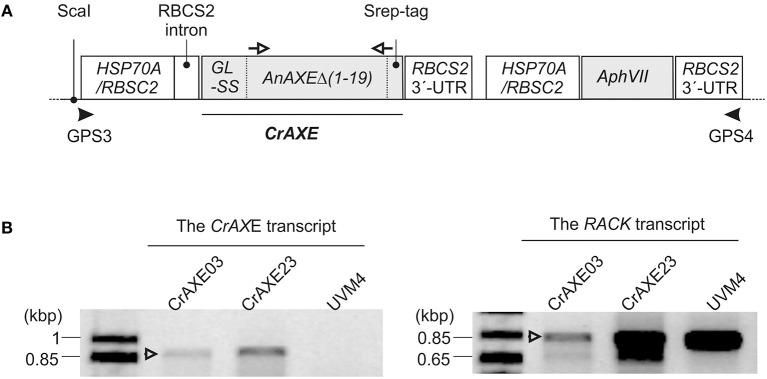
Generation of *C. reinhardtii* transgenic strains expressing the *CrAXE* transcript. **(A)** Schematic illustrations of the gene constructs. The following non-translated genetic elements were included in order to enhance transcription and translation of the recombinant protein: the HSP70A/RBSC2 promoter, the RBCS2 intron, and the RBCS2 3′ UTR, as previously described in Ramos-Martinez et al. (2017). GL-SS indicates the signal sequence derived from gametolysin of *C. reinhardtii* as described in the afore-mentioned reference and is fused to the truncated AnAXE lacking its native signal sequence and a strep tag for detection of the recombinant protein. Primers used for genotyping by PCR and gene specific primers use for RT-PCR are shown by black arrowheads and open arrows, respectively. **(B)** An agarose gel electrophoretic analysis of RT-PCR products derived from the transgenic strains CrAXE03 and CrAXE23. The binding sites of oligonucleotide primers used for RT-PCR analysis are indicated as white arrows in panel A. The expected product size is 859 bp. As a loading control, amplification of a house-keeping gene, *RACK*, was performed.

In order to circumvent uses of NaOH and to establish an eco-friendly and self-sustained growth system, we engineered *C. reinhardtii* to secrete an AXE enzyme for enzymatic hydrolysis of biomass acetylesters. An AXE from the fungus *A. nidulans* (AnAXE), belonging to the carbohydrate esterase family 1 in Carbohydrate Active Enzyme database (www.cazy.org), was chosen for expression in *C. reinhardtii*. This enzyme has been successfully expressed in a broad range of heterologous hosts (Bauer et al., 2006; Pogorelko et al., 2013; Mai-Gisondi et al., 2015). The codon usage of the *AnAXE* gene was optimized according to the genome codon usage of *C. reinhardtii* (Puigbò et al., 2007). The native signal sequence was predicted to be within the first 19 acid residues of the AnAXE protein by *in silico* analysis using SignalP (http://www.cbs.dtu.dk/services/SignalP/) (Petersen et al., 2011) and was replaced by the signal sequence of gametolysin from *C. reinhardtii* to ensure efficient secretion of the recombinant protein (Ramos-Martinez et al., 2017). The design of the expression gene construct, *CrAXE*, is shown in Figure 3A. The *C. reinhardtii* UVM4 strain was used for transformation because of its advantages in reduced gene silencing and high heterologous gene expression (Neupert et al., 2009).

Following transformation with the DNA construct and selection in the presence of paromomycin, 288 colonies were screened by PCR for each construct for the presence of the full-length construct (Figure 3A). The presence of the entire expression cassette, spanning the promoter and the resistance gene, in the genome was confirmed for 158 independent colonies. The expression of the *CrAXE* transcript in 24 transgenic strains, and two transgenic strains, CrAXE03 and CrAXE23, were shown to express the *CrAXE* transcript (Figure 3B).

### Secretion of the CrAXE Enzyme From Transgenic *C. reinhardtii* Strains

**Figure 4:**
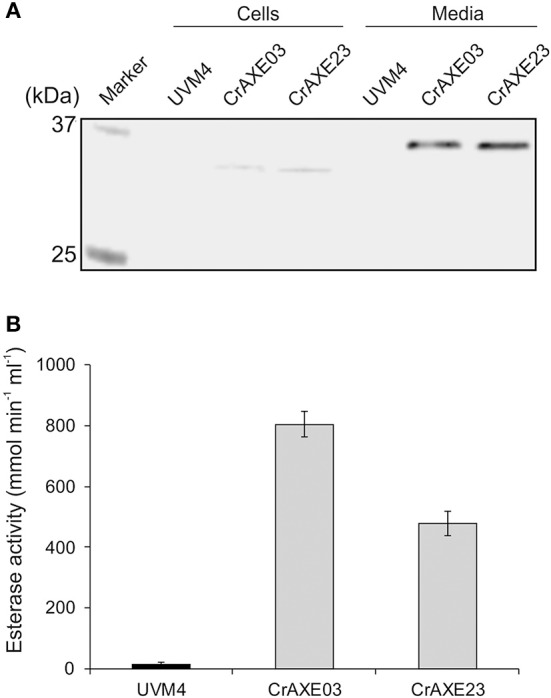
Secretion of the CrAXE protein by the transgenic strains of *C. reinhardtii*. **(A)** Immunoblot analysis of cell lysates (Cells) and cell-free media fractions (Media). **(B)** Acetylesterase activity in cell-free media fractions. The UVM strain and the transgenic strains CrAXE03 and CrAXE23 were grown for 4 days in TAP media under the standard growth conditions. Two milliliters of each culture were centrifuged and the cell pellet and the media fraction were recovered. For immunoblotting, the cell pellet was resuspended in 200 μL of water. Proteins in the media fraction were precipitated by cold acetone and resuspended in 200 μL of water. The equal volume of each material was loaded on a SDS-PAGE gel and immunoblotting was performed using a monoclonal anti-strep antibody. Activity assays were performed directly for the media fraction using 4-nitrophenyl acetate.

Expression of the CrAXE protein was tested for the transgenic strains CrAXE03 and CrAXE23 alongside the UVM4 strain as a control. Four-day cultures of these strains were separated into cells and cell-free media and immunoblotting was performed using a strep-tag antibody. Both transgenic strains showed a strong band at ~35 kDa in the media fraction and a very faint band at ~33 kDa in the cell fraction (Figure 4A). As expected, the UVM4 did not show any detectable products. The expected molecular weight of CrAXE following cleavage of the gametolysin signal sequence is 33.4 kDa. But, as previously reported, *C. reinhardtii* appears to perform yet uncharacterized post-translational modifications, causing increases of apparent molecular weights by ca. 2 kDa (Rasala et al., 2012; Ramos-Martinez et al., 2017). Thus, it was concluded that the 35 kDa product in the media fraction represents the secreted CrAXE protein with a post-translational modification and the 33 kDa product in the cell fractions represents nascent and unmodified CrAXE protein *en route* for secretion.

Acetylesterase activity in cell-free media fractions was measured as previously described (Chung et al., 2002). The transgenic strains CrAXE03 and CrAXE23 showed acetylesterase activity of 800 and 478 μmol min^−1^ mL^−1^, respectively (Figure 4B). The difference between the two lines may be caused by a difference in terms of genome integration sites and/or a number of integrated transgene (Eichler-Stahlberg et al., 2009; Ramos-Martinez et al., 2017). The media fraction derived from the UVM4 strain showed a minor activity close to the detection limit. Taken together, these results demonstrate that the CrAXE03 and CrAXE23 strains secrete active CrAXE enzyme into media.

### Characterization of the Transgenic *C. reinhardtii* in TAP Media

**Figure 5:**
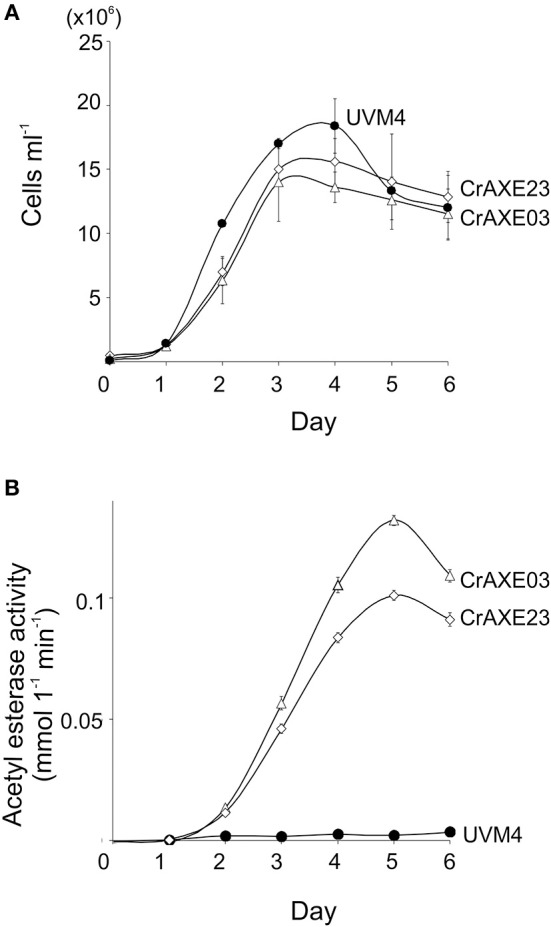
Characterization of the transgenic strains CrAXE03 and CrAXE23. **(A)** Growth curves. **(B)** Acetylesterase activity in the media as a function of time (days). Three independent cultures were analyzed for each genotype. Standard errors are shown. Two-way ANOVA showed that the growth curves and acetylesterase activities between the UVM4 strains and the transgenic strains (CrAXE02 and CrAXE23) were significantly different (*P* < 0.05).

Growth of the transgenic strains CrAXE03 and CrAXE23 were investigated alongside the UVM4 strain. The transgenic strains grew slower than the UVM4 strain by day 2 (one-way ANOVA, *P* < 0.05), but they reached similar cell counts to that of the UVM4 strain by day 3 (Figure 5A). Thus, the production of the CrAXE protein imposes a temporal burden for the growth of *C. reinhardtii* during the early growth phase but the transgenic lines can reach comparable cell counts as that of the UVM4 strain in the late and stationary growth phases. Acetylesterase activity in the culture media of the transgenic lines increased as the cell counts increased, with the CrAXE03 strain showing higher activity than the CrAXE23 strain (ANOVA, *P* < 0.05, Figure 5B). In contrast, the UVM4 strain showed activity that was marginally above the detection limit (Figure 5B).

### Heterotrophic Growth of *C. reinhardtii* Using Wheat Straw Biomass

**Figure 6:**
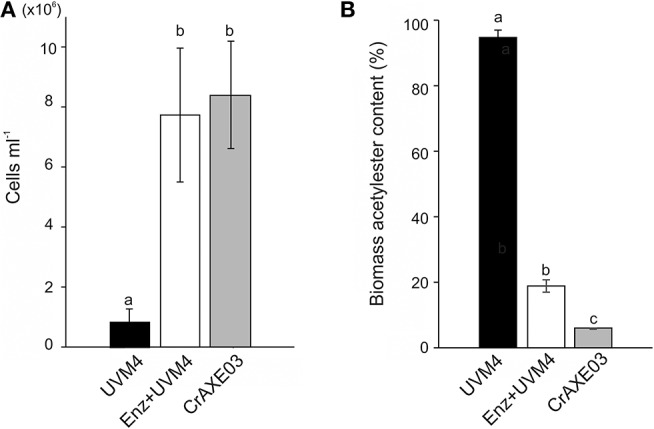
Cultivation of the UVM4 strain and the transgenic strain CrAXE03 of *C. reinhardtii* in the presence of wheat biomass. **(A)** Cell numbers. **(B)** Acetylester content in the wheat straw biomass. The strains were cultivated in the minimal medium containing wheat biomass (MM+Biomass) for 10 days. “Enz+” indicates that a commercial acetylxylan esterase at the concentration of 2 U mL^−1^ was added at the start of cultivation. a, b, and c indicate statistically significant difference (*P* < 0.05).

We then tested if the CrAXE03 strain could grow heterotrophically in MM+Biomass. The CrAXE03 line increased cell counts by eight-folds in the MM+Biomass media in the dark, whereas the UVM4 strain showed only marginal growth under the same conditions (Figure 6A). When the UVM4 strain was supplemented with a commercial acetylxylan esterase at the final concentration of 2 U mL^−1^, it was able to grow to the level similar to that of the CrAXE03 strain. Acetylester contents in biomass materials were determined at the end of the cultivation period (day 10) and compared to the level detected in untreated biomass. A small decrease of acetylesters, by 6%, was found for the biomass treated with the UVM4 strain, possibly due to a basal level of non-specific esterase activities that were observed for the UVM4 culture (Figure 4B). A significantly larger reduction, by 81%, was observed when the UVM4 culture was supplemented with the commercial acetylxylan esterase (Figure 6B). A greater reduction, by 96%, of acetylester contents was observed for the biomass treated with the CrAXE03 strain (Figure 6B). Taken together, these results demonstrate that secretion of an AXE enables *C. reinhardtii* to sequester acetate from acetylesters in wheat straw biomass and assimilate it for growth.

## Discussion

In this study, we showed that secretion of an AXE by *C. reinhardtii* enabled the microalga to utilize acetylesters in wheat straw as a carbon source. Notably, non-engineered *C. reinhardtii* strains (the wild type, the FUD16 strain, and the UVM4 strain) were not able to grow under the same conditions, indicating that recalcitrance of wheat straw hinders assimilation of cellulose (Pauly and Keegstra, 2010). Direct utilization of lignocellulosic biomass has been reported for *A. protothecoides* UTEX 25, likely through assimilation of carbohydrates in the biomass (Vogler et al., 2018), but assimilation of acetylesters has not been. Several industrially important microalga, such as *Chlorella vulgaris* and *Haematococcus pluviaris*, utilize acetate as an organic carbon source (Kobayashi et al., 1993; Estévez-Landazábal et al., 2013). Thus, the approach presented in this study could be applied for a broader range of microalgae, paving a path toward more robust cultivation of microalga.

It is worth noting that treatment of wheat straw biomass by AXE-secreting *C. reinhardtii* caused decreases of acetylesters content in the biomass. It is widely known that acetylesters in lignocellulosic biomass impede saccharification of lignocellulose and acetates released from acetylesters significantly inhibit fermentation of saccharification products by microorganisms like yeast (Casal et al., 1996; Helle et al., 2003; Selig and Himmel, 2009). Notably, a reduction of 20% in the acetate concentration after biomass pretreatment would result increases in the ethanol yields by about 10% and corresponding reduction in the ethanol price (Klein-Marcuschamer et al., 2010). Several approaches have been exploited to solve the acetylester/acetate issues, including extraction by organic solvents (Aghazadeh and Engelberth, 2016) and genetic engineering of plants for reducing contents of acetylation (Lee et al., 2011; Manabe et al., 2011, 2013; Pogorelko et al., 2013; Xiong et al., 2013; Ratke et al., 2015; Schultink et al., 2015). While these approaches are beneficial, they also suffer from negative environmental impacts due to usage of chemical solvents or regulatory restrictions imposed on agricultural cultivation of genetically-modified plants. Thus, direct application of the engineered *C. reinhardtii* culture to lignocellulosic biomass could potentially offer an eco-friendly and flexible way for reducing biomass acetylester contents, thereby improving renewable biofuel productions.

For advancing the present approach to industrial applications, some issues need to be resolved, particularly those relating to host strains. The experiments in this study were carried out using the *C. reinhardtii* UVM4 strain because it offers low gene silencing activity (Neupert et al., 2009). But this strain also lacks the cell wall. The lack of the cell wall may be advantageous because the secreted protein is directly deposited into the culture media without having to pass through the cell wall matrix as in the case of the wild type, but it could also render the cell more prone to stresses imposed by mixing of cultures (i.e., shear stress) and possible toxic compounds in media. These aspects are yet to be rigorously examined in large-scale growth facilities relevant for industrial applications. As for uses of industrially important microalgae as the host organisms, such as those mentioned above, establishment of efficient transformation protocols and genetic tools are prerequisite and is still to come. Lastly and not least, genetic instability is a key issue and is widely observed for nuclear genome transformation in *C. reinhardtii*, where expression of transgenes typically diminishes over generations. Unfortunately, underlining causes of genetic instability are not fully understood. Thus, future research opportunities in microalgal biotechnology are abundant and will likely cover wide topics from carbon sources, development of robust transformation and genetic tools, optimization of growth facility, and discoveries of ways to overcome genetic instability.

## Data Availability

All datasets generated for this study are included in the manuscript and the supplementary files.

## Author Contributions

EMR-M, LF, and YS conceived the study. EMR-M, LF, and MKR performed experiments. EMR-M, LF, MKR, and YS performed data analysis. EMR-M and YS prepared the original draft. All authors contributed to the final manuscript.

### Conflict of Interest Statement

The authors declare that the research was conducted in the absence of any commercial or financial relationships that could be construed as a potential conflict of interest.
