# Supplementary material for: Secretion of Acetylxylan Esterase From Chlamydomonas reinhardtii Enables Utilization of Lignocellulosic Biomass as a Carbon Source
Source: Front Bioeng Biotechnol. 2019 Feb 28;7:35. doi: 10.3389/fbioe.2019.00035 (PMC6403119; doi:10.3389/fbioe.2019.00035)
Supplement: Supplementary file 1 [file Data_Sheet_1.docx]

Supplementary Material

Secretion of acetylxylan esterase from *Chlamydomonas reinhardtii* enables utilization of lignocellulosic biomass as a carbon source First

Erick Miguel Ramos-Martinez, Lorenzo Fimognari, Maria K. Rasmussen, Yumiko Sakuragi

*** Correspondence:** Corresponding Author: ysa@plen.ku.dk

# Supplementary Data

**The nucleic acid sequence of the CrAXE gene construct**

ATGTCGCTGGCGACGCGGCGCTTCGGCGCCGCAGCGGCGCTTCTAGTCGCCGCATGCGTGCTGTGCACAGCTCCTGCGTGGGCCGTGAAGCTCCAGTACCTGCTGTCCATCCTGCTCTACGCGTACTCCTGCACGGCCCTCATGCTGGACCGCCGCGACCCGACGCCCGGGCAGCTCTCCCAGGTGACCGACTTTGGGGATAACCCGACCAACGTCGGGTTTTACATTTACGTGCCCCAGAACCTGGCCTCGAACCCGGCCATCATTGTGGCTATTCATTACTGCACGGGCACGGCCCAGGCGTACTACTCCGGGACCCCCTACGCGCAGTACGCTGAGACGTACGGGTTTATCGTCATCTACCCCGAGTCCCCCTACTCGGGGACGTGCTGGGACGTCTCCAGCCAGAGCACGCTCACGCACAACGGGGGGGGTAACTCGAACTCGATTGCGAACATGGTCGACTGGACGATTAACCAGTACAACGCGGACGCCAGCCGGGTCTACGTCACGGGCACGTCGAGCGGCGCGATGATGACCAACGTCATGGCTGCTACCTACCCCAACCTGTTTGCCGCGGGGATCGCGTACGCGGGGGTGCCCGCTGGGTGCTTCTACAGCGAGGCGAACGTCGAGGACCAGTGGAACAGCACCTGCGCTCAGGGGCAGAGCATCTCCACGCCTGAGCATTGGGCCCAGATCGCTCAGGCTATGTACTCCGGCTACGAGGGCTCCCGCCCCAAGATGCAGATCTACCACGGGTCCGCGGACGCGACGCTCTACCCCCAGAACTACTACGAGACGTGCAAGCAGTGGGCTGGCGTCTTCGGCTACAACTACGACTCGCCCCAGGAGGTCCAGAACGATACGCCTGTGGCTGGCTGGGCCAAGACCATTTGGGGTGAGAACCTCCAGGGTATCCTGGCCGACGGCGTCGGGCACAACATCCAGATCCAGGGTGAGGAGGATCTGAAGTGGTTCGGTTTTACGAGCTGGAGCCACCCGCAGTTCGAGAAGTAA
